# Anesthetic management of a patient with congenital insensitivity to pain with anhidrosis by coadministration of remifentanil

**DOI:** 10.1186/s40981-018-0208-8

**Published:** 2018-10-03

**Authors:** Yoko Takeuchi, Yoshihito Fujita, Takeshi Shimomura, Shuji Kurokawa, Hiroki Noguchi, Yoshihiro Fujiwara

**Affiliations:** 1 Department of Anesthesiology, Aichi Medical University School of Medicine, 1-1 Yazako Karimata, Nagakute, Aichi 480-1195 Japan; 2 Department of Anesthesiology, JA Aichikoseiren Konan Kosei Hospital, 137 Oomatubara Takaya, Konan, Aichi Japan

**Keywords:** Congenital insensitivity to pain with anhidrosis, Insensitivity, Anhidrosis, Remifentanil, Safety

## Abstract

**Background:**

Congenital insensitivity to pain with anhidrosis (CIPA) is a rare autosomal recessive disease characterized by unexplained fever, systemic insensitivity to pain, anhidrosis, and mental distress. Anesthetic management is challenging because autonomic dysfunction can induce perioperative complications. Only a few reports of anesthetic management of CIPA patients have been published. We herein present a case of successful management of the same patient on two occasions using small doses of fentanyl and remifentanil.

**Case presentation:**

A 37-year-old man with CIPA underwent two orthopedic operations. We were able to balance the dose of remifentanil to avoid the extremes of hyperalgesia when the dose is too low and shivering when the dose is too high.

**Conclusion:**

To our knowledge, no reports have described the anesthetic management of CIPA patients with remifentanil. We consider anesthetic management with coadministration of remifentanil to be potentially useful for such patients.

## Background

Congenital insensitivity to pain with anhidrosis (CIPA) is a rare disease classified as hereditary sensory and autonomic neuropathy (HSAN) type IV [1, 2] according to Dyck et al. [1], who categorized congenital hyposensitivity to pain into five different types of HSANs. The human TRKA gene encodes the receptor tyrosine kinase for nerve growth factor, and its mutation is responsible for CIPA [3]. CIPA is characterized by loss of pain and thermal sensation accompanied by mental distress. Other sensory modalities such as touch, pressure, and vibration are not affected. Insensitivity to pain leads to bone fractures, burns, and self-mutilation of the tongue, lips, or fingers. Repeated injuries attributable to pain insensitivity may require repeated surgeries.

Anesthetic management for these patients is challenging because their autonomic dysfunction may induce perioperative complications, such as hyperthermia or hypothermia, tachycardia or bradycardia, and hypertension or hypotension. In addition, some sensory modalities remain intact and analgesia is still needed when the patient reacts to surgical stimuli. A few reports of the anesthetic management of patients with CIPA have been published [4, 5]. A retrospective analysis of 35 patients [4] demonstrated that anesthesia was induced with propofol (71%), muscle relaxants (27%), and fentanyl (8%). Anesthesia was maintained with propofol (29%), volatile anesthetics (28%), nitrous oxide (46%), muscle relaxants (6%), and fentanyl (2%). Complications included hyperthermia (> 37.5 °C) (3 patients), aspiration (2 patients), cardiac arrest (1 patient), and bradycardia (10 patients). In another report of two HSAN IV patients [5], anesthesia was induced with thiopental or volatile anesthetics and was maintained with volatile anesthetics and fentanyl or oxymorphone. In this instance, there were no perioperative complications. To our knowledge, there have been no reports to date of anesthetic management of these patients by administering remifentanil. Here, we report the successful anesthetic management of a 37-year-old man with CIPA by coadministration of remifentanil during two consecutive operations.

## Case presentation

**Fig. 1:**
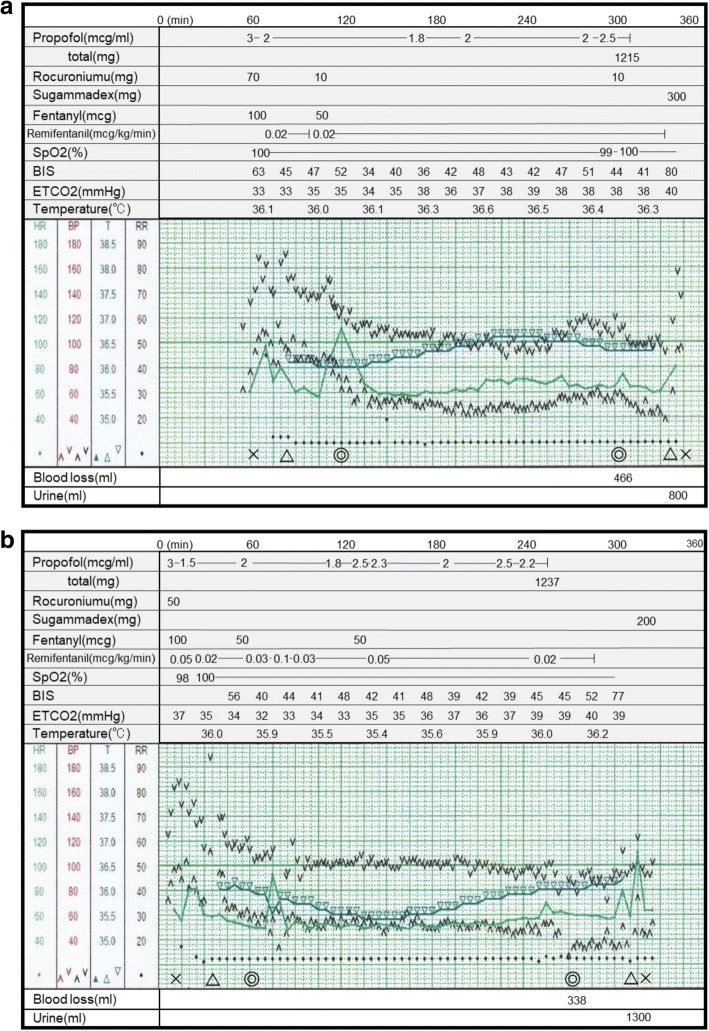
Anesthetic charts of the first operation (**a**) and the second operation (**b**). X represents the start and finish of anesthesia, △ represents the intubation and extubation, and ◎ represents the start and finish of surgery. SpO_2_, O_2_ saturation of the pulse; BIS, bispectral index; ETCO_2_, end-tidal CO_2_; min, minutes

Informed consent for scientific publication was obtained. A 37-year-old man (153 cm, 69 kg) with CIPA underwent an operation for posterior spinal fusion to treat thoracic spondylotic myelopathy. His sensory deficits included hyposensitivity to superficial and deep visceral pain, thermal hyposensitivity, and he have mild mental destress, unimpaired touch, and pressure sensitivity. Because of self-mutilation such as tongue or finger biting, his mouth and limbs were deformed; however, he lived independently and had a job. Autonomic imbalance was not remarkable. All members of his family did not have any symptoms of this disease. Genetic test was undergone and diagnosed with CIPA (HSAN IV). Presenting with symptoms of gait disorder and numbness of the lower limbs, he was diagnosed with thoracic spondylotic myelopathy. He had previously undergone no operations under general anesthesia. Laboratory tests were normal. In the first operation, we monitored electrocardiography, non-invasive blood pressure measurements, oxygen saturation, end-tidal CO_2_, bispectral index (BIS), and body temperature via rectal probe. Anesthetic induction was applied with intravenous propofol (3 μg/ml of target control infusion [TCI]), fentanyl (100 μg), and rocuronium (70 mg). After intubation and at the time of skin incision, the patient’s blood pressure and heart rate increased (Fig. 1a). We administered 50 μg of fentanyl. Anesthesia was maintained with propofol (1.8–2.5 μg/ml of TCI) and remifentanil (0.02 μg/kg/min) to keep the BIS between 40 and 60. In the middle of the operation the blood pressure and heart rate increased slightly while remaining within the normal range. The body temperature was maintained between 36.0 and 36.6 °C using a warming blanket with hot-air and regulation of operating room temperature. After extubation, the patient felt discomfort in the throat. The patient did not receive any opioids after the operation, and his postoperative course was uneventful. However, after the surgery, he experienced bladder and rectal disturbance. Spinal cord compression was presumed to have occurred, and laminectomy was planned.

In the second operation (Fig. 1b), the anesthetic management course was almost the same. Anesthetic induction was applied with intravenous propofol (3 μg/ml of TCI), fentanyl (100 μg), and rocuronium (50 mg). After induction, we adjusted the propofol and remifentanil to maintain a stable circulatory status. After operation starting body temperature was decreased to 35.4 °C. Using warming blanket with hot-air, temperature was increased at 36.2 °C. After extubation, the patient reported no sore throat, wound pain, or shivering. He did not receive any opioids after the operation, and no perioperative complications were noted. After surgery, he was discharged and continued with daily life using a wheelchair and indwelling urinary catheter.

## Discussion

We undertook two consecutive successful managements of the same CIPA patient by using a small dose of fentanyl and remifentanil without significant alterations of blood pressure, heart rate, or temperature. Because patients with CIPA have pressure sensitivity despite a lack of pain sensitivity, we administered an analgesic agent, i.e., a small dose of fentanyl and remifentanil, to obtain a stable anesthetic condition. To our knowledge, thus far, no reports have described the anesthetic management of patients with CIPA with coadministration of remifentanil.

There are reports of sympathetic nervous response to surgical stimulation and tracheal intubation as measured by the plasma catecholamine levels, whereby catecholamine levels were not elevated in the absence of analgesic drugs [6–8]. However, despite the lack of pain sensitivity, perceptions of touch, pressure, and vibration are more sensitive [2]. Therefore, some patients require fentanyl for airway manipulation [4, 5]. In our patient, the blood pressure and heart rate increased after intubation and at the time of skin incision. In addition, in the middle of the first operation, the blood pressure and heart rate increased slightly, although they remained within the normal range. In the second operation, we added a small dose of fentanyl and adjusted remifentanil to obtain a stable circulatory status. We speculated that these were reactions to the pressure of surgical stimuli, which disappeared after surgery; therefore, the use of a short-acting opioid (remifentanil) may be of benefit in adjusting the circulatory condition during such pressure stimuli.

In spite of body temperature dropping to 35.4 °C, it was relatively well-controlled during anesthesia using a warming blanket and regulation of operating room temperature. Mild hypothermia is one of the reported complications of anesthetic management in CIPA patients [4, 5]. Use of a thermal blanket and regulation of ambient temperature for our patient led to no perioperative complications related to body temperature. Although remifentanil is associated with an increased incidence of post-anesthetic shivering [9], this complication did not occur in our patient probably because CIPA patients have impaired thermoregulation. Shivering is frequently observed as side effect of remifentanil infusion. At the same time, shivering is the autonomic defense system against low body temperature. Absence of shivering reaction may not always provide benefit to this patient. CIPA patients lack primary afferents and sympathetic postganglionic neurons. However, the mechanism that more dosages of remifentanil induce a higher rate of shivering is not unclear [9]. Not only nerve of the autonomic defense but also muscle itself might be related. In any case, in our patient, shivering did not occur using a small dose of remifentanil.

Regarding other complications of anesthetic management of these patients, postoperative nausea and vomiting (PONV), aspiration, and cardiovascular instability including cardiac arrest have been documented [4, 5]. Patients with CIPA also have autonomic nervous system abnormalities, which may lead to PONV or aspiration [4]. In our case, PONV was not observed. We did not use analgesic drugs such as acetaminophen and nonsteroidal anti-inflammatory agents to treat postoperative pain. The patient felt discomfort in the throat but no pain. Although remifentanil can lead to acute tolerance and hyperalgesia [10], a patient with CIPA may avoid this complication. As CIPA is an infrequent disease for which it is difficult to estimate the sensory responses, further study of the safety of anesthesia for patients with CIPA is warranted.

In conclusion, two anesthetic managements of the same patient with CIPA using coadministration of remifentanil were successful, with no perioperative complications.

## Abbreviations

BIS: Bispectral index
CIPA: Congenital insensitivity to pain with anhidrosis
ETCO_2_: End-tidal CO_2_
HSAN: Hereditary sensory and autonomic neuropathy
PONV: Postoperative nausea and vomiting
SpO_2_: O_2_ saturation of the pulse
TCI: Target controlled infusion
